# Geometric morphometrics reveals sex-differential shape allometry in a spider

**DOI:** 10.7717/peerj.3617

**Published:** 2017-07-26

**Authors:** Carmen Fernández-Montraveta, Jesús Marugán-Lobón

**Affiliations:** 1 Departamento de Psicología Biológica y de la Salud, Universidad Autónoma de Madrid, Madrid, Spain; 2 Departamento de Biología, Universidad Autónoma de Madrid, Madrid, Spain; 3 Dinosaur Institute, Natural History Museum of Los Angeles County, Los Angeles, CA, USA

**Keywords:** Sexual size dimorphism, Allometry, Sexual selection, Spiders, *Donacosa merlini*, Doñana

## Abstract

Common scientific wisdom assumes that spider sexual dimorphism (SD) mostly results from sexual selection operating on males. However, testing predictions from this hypothesis, particularly male size hyperallometry, has been restricted by methodological constraints. Here, using geometric morphometrics (GMM) we studied for the first time sex-differential shape allometry in a spider (*Donacosa merlini*, Araneae: Lycosidae) known to exhibit the reverse pattern (i.e., male-biased) of spider sexual size dimorphism. GMM reveals previously undetected sex-differential shape allometry and sex-related shape differences that are size independent (i.e., associated to the y-intercept, and not to size scaling). Sexual shape dimorphism affects both the relative carapace-to-opisthosoma size and the carapace geometry, arguably resulting from sex differences in both reproductive roles (female egg load and male competition) and life styles (wandering males and burrowing females). Our results demonstrate that body portions may vary modularly in response to different selection pressures, giving rise to sex differences in shape, which reconciles previously considered mutually exclusive interpretations about the origins of spider SD.

## Introduction

Sexual dimorphism (SD), defined as size or morphological differences between the sexes (Hedrick & Temeles, 1989) is a large source of phenotypic variation in animals. As a relevant topic in evolutionary biology ever since Darwin (Lande, 1980; Lande & Arnold, 1985; Hedrick & Temeles, 1989; Fairbairn, Blanckenhorn & Szekely, 2007), the evolution of SD is known to result from genetic correlations between the sexes and sex-differential selection pressures, particularly natural selection related to reproductive sex roles or life styles and sexual selection, related to mate competition (Lande, 1980; Lande & Arnold, 1985; Hedrick & Temeles, 1989; Cox, Skelly & John-Alder, 2003; Foellmer & Fairbairn, 2005a; Fairbairn, Blanckenhorn & Szekely, 2007). Hypotheses testing, however, remains controversial, as the aforementioned mechanisms are not necessarily mutually exclusive and several mechanisms may be operating at the same time (Hedrick & Temeles, 1989).

As unique systems in the analysis of the ultimate causes of SD (Foellmer & Moya-Laraño, 2007), spiders are a widely featured model system in this controversy. Overall, spiders follow the arthropod female-biased pattern of sex differences in size, but spider sexual size dimorphism (SSD) actually spans from moderately male-biased (Alderweireldt & Jocqué, 1991a; Schutz & Taborsky, 2003; Aisenberg, Viera & Costa, 2007) to the most extremely female-biased terrestrial patterns (Foellmer & Fairbairn, 2005a; Vollrath, 1998). However, the prevalent explanations about the evolutionary origins of spider SD focus on a single mechanism, namely sexual competition among males (Foellmer & Moya-Laraño, 2007). Male size usually predicts the outcome of male–male interactions (Elias et al., 2008; McGinley, Prenter & Taylor, 2015), and males are under directional selection for overall larger size even in extremely female-biased spider taxa (Foellmer & Fairbairn, 2005a). As far as interference competition relaxes when population densities are low, selection may yet favor male morphologies best advantageous at scramble competition (Foellmer & Moya-Laraño, 2007). For instance, relatively smaller males are better at climbing to reach females (Moya-Laraño, Halaj & Wise, 2002), whereas spider SSD is more accentuated when male mortality increases because of the sex-differential costs of travelling in search for females (Elgar, 1991; Vollrath & Parker, 1992; Walker & Rypstra, 2001; Moya-Laraño, Halaj & Wise, 2002; Walker & Rypstra, 2003; Brandt & Andrade, 2007). However, spider SSD fails to follow a common trend of phenotypic variation resulting from sexual selection, namely the higher variation of male size over evolutionary times. This trend would lead to a reduced SSD the larger the body size in such a female-biased taxon (i.e., Rensch’s Rule (Rensch, 1959)). However, spider female size tends to vary more than male’s within and across taxa (Abouheif & Fairbairn, 1997), which might be consistent with a prevalent role of fecundity selection in the evolution of spider SSD (Head, 1995; Coddington, Hormiga & Scharff, 1997).

Selection pressures acting differently on particular male and female body parts are likely to produce different patterns of allometric growth (Zelditch, Swiderski & Sheets, 2012), leading to sex differences in shape and sexual shape dimorphism (Fairbairn, 2007; Szekely, 2007). Furthermore, sexually selected traits have been long suspected to show positive static allometry, as larger individuals are expected to exhibit disproportionally larger traits (Kodric-Brown, Sibly & Brown, 2006), and sex-differential allometric variation could hence indicate a role for sexual selection in the evolution of particular traits. However, the analysis of spider shape and shape allometry has not attracted much attention, likely because of methodological constraints. For instance, as in many other groups (Stuart-Fox, 2009; Cabrera, Scrocchi & Cruz, 2013; Roitberg et al., 2015), spider SSD is usually analyzed either from single linear body measurements (Jakob, Marshall & Uetz, 1996; Aisenberg, Viera & Costa, 2007; Aisenberg et al., 2010) or from linear combinations of them obtained from multivariate statistical techniques, essentially principal components analysis (PCA) (Prenter, Montgomery & Elwood, 1995; Foellmer & Fairbairn, 2005a, 2005b), which is limited to describe shape as residual deviations from size (Mosimann, 1970). Today, landmark-based geometric morphometrics (GMM) stands among the suite of new analytical tools which have been successfully applied to the analysis of sexual shape dimorphism (O’Higgins & Collard, 2002; Pretorius, Steyn & Scholtz, 2006; Kaliontzopoulou, Carretero & Llorente, 2007; Bonnan, Farlow & Masters, 2008; Benítez, 2013). In spite of the great potential of GMM for the evaluation of shape and sex-differential shape allometry, however, the method has not been ever applied to the study of spider sexual shape dimorphism and shape allometry, with the exception of studies on shape variation in key characters of spider taxonomy, such as genital structures (Crews, 2006; Costa-Schmidt & de Araujo, 2010).

Here, we applied GMM to the analysis of body shape allometry and sexual shape dimorphism in a wolf spider (*Donacosa merlini* Alderweireldt & Jocqué, 1991a, Araneae, Lycosidae), known to unambiguously exhibit male-biased SSD on the basis of traditional morphometrics (Alderweireldt & Jocqué, 1991b). We measured spider size and shape at several locations, covering the whole range of habitat variation in the known species distribution area. *D. merlini* shows sex differences in reproductive roles and life styles; mature males are the roving sex and search for mates, whereas females are obligate burrowers during their whole lives and remain inside their burrows for cocoon production and spiderling hatching, caring for cocoons and spiderlings before dispersal. Particular body parts may relate differentially to male and female fitness: spider opisthosoma structures relate to vegetative tasks, including reproduction (Foelix, 1996), and larger opisthosomas enable increased energy and egg storage and production. Therefore, fecundity selection should tend to favor the evolution of female-biased opisthosomas or total body length (Preziosi et al., 1996; Head, 1995). On the contrary, spider prosoma structures relate to locomotion, food intake and integrative nervous functions (Foelix, 1996), and arguably larger carapaces could improve resource holding potential, trophic efficiency or spider mobility. If natural selection drives the evolution of SD, we expected sex differences in the allometric growth of prosoma and opisthosoma structures (relatively larger female opisthosomas and more robust male prosomas), leading to size-independent sexual shape dimorphism. Moreover, if sexual selection drives the evolution of sexual shape differences, we expected sex-differential carapace shape allometry.

## Methods

### The species

*Donacosa merlini* is a burrowing, medium-sized wolf spider the distribution of which is restricted to the sandy coastal areas surrounding Doñana in Southwestern Spain, where it inhabits a variety of habitats, ranging from grasslands to xerophytic scrublands (Fernández-Montraveta & Cuadrado, 2008). *D. merlini* is the only known worldwide representative of the genus (Alderweireldt & Jocqué, 1991a, 1991b), and exhibits singular life history traits: mating is delayed compared to closely related wolf spiders and takes place during the late autumn; males mature later than females and reach larger maturation sizes. Spiderlings hatch in spring and mature after a relatively long post-embryonic development of 15–20 months under laboratory conditions (Fernández-Montraveta, Moya-Laraño & Cuadrado, 2004). The species is semelparous, and females exhibit maternal care; cocoon and spiderling carrying is mostly restricted to the female burrow (Fernández-Montraveta & Cuadrado, 2008).

### Experimental setup

During the mating season, we captured mature spiders at four different field sites representing almost the entire range of habitats at which *D. merlini* is known to occur in the Doñana area (field project number 022/2007). At this time of the year, only mature females and juveniles are found inside burrows; we captured females by hand following burrow excavation during the day (10:00–17:00 hours) and males after the sunset (19:00–21:00 hours) during mate searching, using headlamps. We only included mature animals in the study, hence returning immature spiders back to their burrows immediately after instar determination by visual inspection. Following capture, we transported spiders to the laboratory for individual measurements. We measured spiders from dorsal pictures of living individuals (Olympus E-300 digital camera); for the pictures, we immobilized spiders inside a plastic bag placed on top of a scaled paper, to avoid any motion and prevent individual damage. To standardize the spider position, we always oriented the animal horizontally and facing to the right. We returned spiders to the field on the day following capture; to reduce predation risk, we placed females back to their reconstructed burrows and males in the vegetation. Finally, to minimize any negative effect of manipulation on populations of this singular and protected species (Verdú & Galante, 2005; Barea-Azcón, Ballesteros-Duperón & Moreno, 2008), we prevented any accidental damage of burrows by marking them and removing all marks when the fieldwork was over. Overall, we captured and measured 178 spiders (97 males and 81 females).

**Figure 1:**
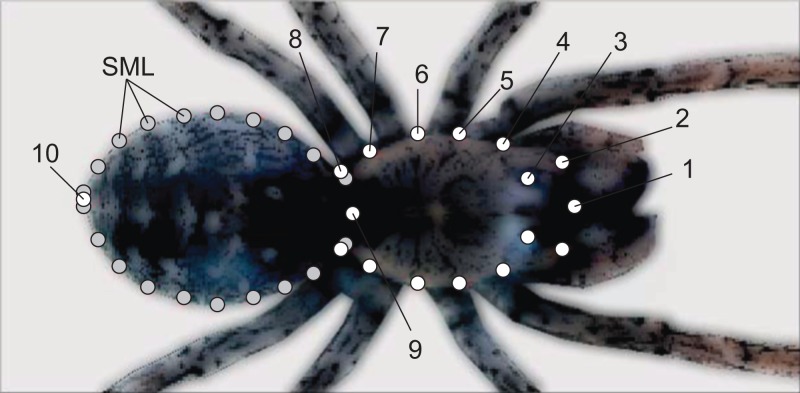
Landmarks (white) and semilandmarks (gray) exemplified on a spider picture.

On each spider picture, we digitized 37 bidimensional landmarks for shape analysis. The spider’s anterior body portion (i.e., prosoma) is covered by two firm plates (dorsal carapace and ventral sternum) and only expands at molting, whereas the posterior opisthosoma is comparatively softer and expands as the spider stores nutrients or develops eggs (Foelix, 1996). On the spider’s carapace, we digitized 17 landmarks and 20 along the outline of the opisthosoma (Fig. 1). The carapace landmarks are all optimal (Type I) (Bookstein, 1991), as their spatial positions are defined on the basis of highly repeatable and unambiguous anatomical locations (i.e., the places at which legs, pedipalps or chelicerae insert at the carapace; see Fig. 1). We spaced the points regularly along the opisthosoma outline, and therefore treated these opisthosoma coordinates as semilandmarks (Zelditch, Swiderski & Sheets, 2012).

To obtain the shape data, landmark configurations were superimposed using the generalized Procrustes method, also known as general Procrustes analysis (Adams, Rohlf & Slice, 2004), based on a generalized least-squares minimization of the distance between corresponding landmarks (Gower, 1975). Landmark configurations are compared by this superimposition, which is achieved by translating, rotating and scaling all configurations to a common reference system (the mean). Shape in this context is the residual mismatch and irreducible distance among homologous landmarks after the complete Procrustes alignment, and is thus “invariant” to (i.e., it does not possess any information about) translation, rotation and scale. To treat the opisthosoma semilandmarks, we used the minimum Procrustes distance sliding method (Bookstein, 1997), better suited for relatively simple outlines such as those of the opisthosoma at intraspecific levels (Pérez, Bernal & González, 2006). Following the Procrustes fit, we used PCA to summarize the sample shape variation into fewer components. As a proxy for size we used the centroid size of the landmark configurations, which corresponds to the squared root of the sum of the squared distances from each landmark to the centroid (Bookstein, 1991). We calculated two separate shape and size datasets, one including the whole set of landmarks and semilandmarks (i.e., both the carapace and the opisthosoma) which thereby accounted for overall body size and shape, and another excluding the opisthosoma semilandmarks, thereby only capturing the carapace size and shape.

We used the TPSrelw program (v.1.49; Rohlf, 2010) to slide the semilandmarks and MorphoJ (v. 1.6.0_27, (Klingenberg, 2011)) to perform all subsequent analyses, including landmark superimposition. MorphoJ allows isolating a component of shape that only accounts for symmetric variation out of a bilaterally symmetric configuration of landmarks (Mardia, Bookstein & Moreton, 2000; Klingenberg, Barluenga & Meyer, 2002). The method, informally called “symmetrization”, yields a component of shape variation among individuals in what might be considered a left-right averaging (Klingenberg, Barluenga & Meyer, 2002; Savriama et al., 2012). This helps ignoring any source of variation within the sample due to asymmetry, thus reducing the small yet potential error introduced by, for instance, measuring immobilised animals with a globular morphology and bilateral symmetry such as spiders. Shape allometry was studied using multivariate regression analyses (Monteiro, 1999) based on the pooled within group variance, to control for the effect of sexual size differences on shape variation. For further comparisons between the sexes, we used a discriminant function analysis. For traditional size comparisons, we applied general linear models (sex as the fixed factor) on body size and carapace size. We applied multivariate regressions of the shape variables on centroid size to analyze body and prosoma shape allometry. As a method for verification, such allometric models were compared using univariate general linear models on the obtained regression shape scores, including sex as a fixed factor and size as the covariate (significant sex×centroid size interaction would indicate that the two allometric slopes differ; Klingenberg, 2016). We used R (R Core Team, 2017) for the standard statistical analyses.

## Results

### Shape variation and sexual shape dimorphism

**Figure 2:**
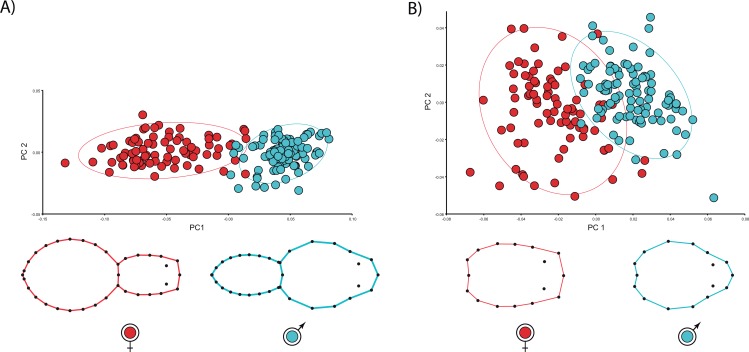
Principal Component Analysis of shape variation. (A) body and (B) carapace shapes. Extreme female and male shapes are polarized at the extreme of PC1 (*x* axis). (A) Males (blue dots) and females (red dots) clearly differ on PC1, which explains most body shape variation (85.7%). (B) Sex differences in carapace shape are also distinct.

We found unequivocal sexual differences in body and carapace shapes. The first two PCs explained 89.1% body shape variance. PC1 (85.7% variance explained) is the dominant dimension of variation and unambiguously yields an ordination related to sexual shape differences (Fig. 2). Females clearly show more globular opisthosomas and laterally flattened and cephalically squared carapaces. Males exhibit much slender opisthosomas and both laterally and cephalically protruding carapaces (Fig. 2A). The ordination was far more dispersed for carapace shape, and the two first PCs only accounted for 58.4% variance. Nonetheless, PC1 (44.5% variance explained) definitely separates male and female shapes (Fig. 2B). Not surprisingly, the CVA clearly finds a statistically predictable sexual shape dimorphism both for the body (*p* < 0.001) and the carapace (*p* < 0.001).

### Allometry and size-independent shape variation

The statistics of both body and carapace sizes revealed significant sex differences; females are 1.1 larger than males for body size. However, sexual size differences revert, and males turn out to be 1.1 times larger than females for carapace size (see Table 1).

**Table 1:** Summary statistics (mean ± 1SE) of size and shape depending on the spider sex (male–female).

Trait	Males		Females	*F*	df	*p* Value
Body size	2.9 ± 0.03		3.1 ± 0.05	14.1	1	<0.001
Carapace size	1.3 ± 0.02		1.2 ± 0.02	14.3	1	<0.001
Body shape	PC1	0.04 ± 1.7 × 10^−3^	−0.05 ± 3.6^−3^	674.2	1	<0.001
	PC2	−0.001 ± 1.12 × 10^−3^	0.001 ± 1.1 × 10^−3^	2.1	1	0.15
	CV1	3.3 ± 0.1	−4 ± 0.1	229.8	1	<0.001
Carapace shape	PC1	0.03 ± 1.19 × 10^−3^	−0.04 ± 2.1 × 10^−3^	777.4	1	<0.001
	PC2	4.02 × 10^−5^ ± 1.88 × 10^−3^	−5.2 × 10^−5^ ± 2.2 × 10^−3^	9 × 10^−4^	1	0.98
	CV1	2.7 ± 0.09	−3.2 ± 0.1	154.3	1	<0.001

**Note:**

We estimated spider shape and size on the basis of the overall body or the carapace landmarks. Shape is described after the Canonical Variate 1 and the two first PCs from a PCA following Procrustes superimposition of individual landmark configurations. Spider size is the centroid size. Significant differences are highlighted.

**Figure 3:**
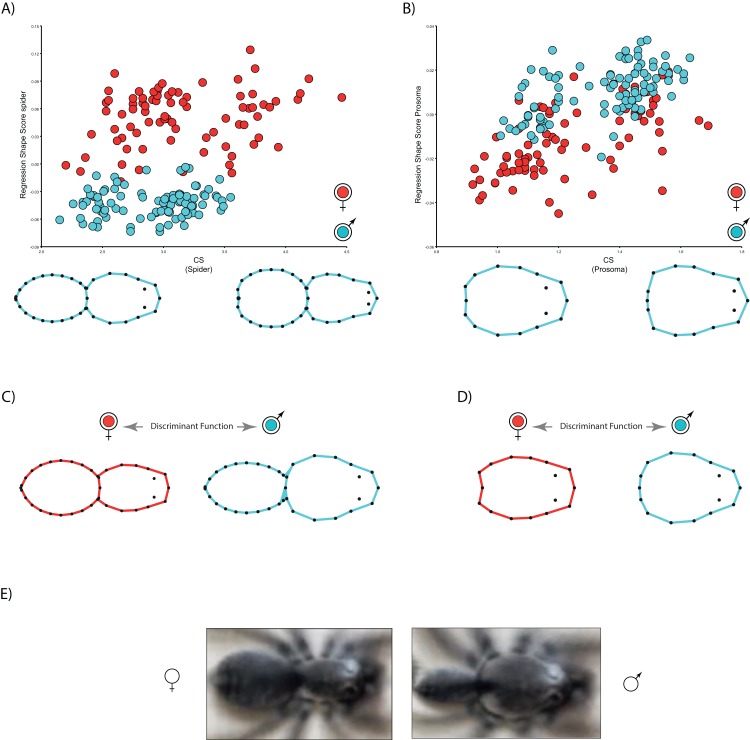
Allometric regressions of spider shape. (A) body and (B) carapace shapes. (C) Histogram with the scores for the leave-one-out cross-validation of Discriminant analysis in body and (D) carapace shapes. (E) Shape differences between females and males shown as image deformations (warps) of the original stock of images computed with the Image Unwarp algorithm using TPSSuper (v.1.15; Rohlf, 2013) (Marugán-Lobón & Buscalioni, 2009; Rohlf, 2002).

The multivariate regressions indicate that spider shape variation is clearly allometric. Interestingly, the allometric slopes are not sexually dimorphic (Body shape: males 0.01, females 0.01; carapace shape: males 0.04, females 0.05), whereas the intercepts (Body shape: males −0.06, females 0.007; carapace shape: males −0.04, females −0.07) are (Table 2; see Fig. 3). The sign of intercept differences revert depending on the particular body part studied (i.e., either whole body or carapace) (see Fig. 3). This notwithstanding, sexual shape dimorphism is size-independent, as sex differences remain when we statistically control for the effect of body size (Table 2). In fact, the PCA on the regression residuals extracts again two independent components, and PC1 explains 67.2% of body and 57.8% of carapace shape variance, respectively; PC1 clearly discriminates between males and females, thus corroborating the size-independent sexual shape dimorphism. This result stands either including, i.e., body shape, or excluding, i.e., carapace shape, the opisthosoma information. Again, relatively slender opisthosomas and carapaces both laterally and cephalically protruding characterize male shape. On the contrary, females exhibit relatively larger and more globular opisthosomas as well as laterally flattened and cephalically squared carapaces. Reinforcing these results, CVA unambiguously discriminates between size-independent male and female shapes (body shape: Procrustes distance 0.09, *p* < 0.001; carapace shape: Procrustes distance 0.06, *p* < 0.0001).

**Table 2:** Results of regression analyses showing the allometric intercepts and slopes of regression scores on size, depending on the body portion considered (body shape, carapace shape).

Trait	Effect	df	*F*	*p* Value
Body shape	Size	1	7.5	<0.01
	Sex	1	646.3	<0.001
	Size × sex	1	0.8	0.4
Carapace shape	Size	1	85.4	<0.001
	Sex	1	109.4	<0.001
	Size × sex	1	0.8	0.4

**Note:**

Significant differences are highlighted.

## Discussion

Our results on shape variation in *D. merlini* reveal new and previously undetected aspects of spider sexual morphological variation. Using shape analysis GMM, we have quantified for the first time that, (1) spider males and females unambiguously differ in body shape, (2) such shape differences relate to two main features: the carapace-to-opisthosoma ratio and the carapace shape, (3) a portion of spider shape variation is allometric, but sexual shape differences are completely independent on size, and (4) the shape allometric intercepts are sexually dimorphic, whereas the slopes—scaling—are not.

The evolution of spider sexual dimorphism has been widely studied (Foellmer & Moya-Laraño, 2007) on the basis of single linear measurements of size, such as the maximum carapace width or the correlated carapace body or leg lengths (Jakob, Marshall & Uetz, 1996; Aisenberg, Viera & Costa, 2007; Aisenberg et al., 2010). Thus far, such empirical evidence supports a primary role of sexual selection operating on male bodies, but hyperallometry of male size or sex-differential shape allometry had been seldom addressed. On the other hand, spider multivariate sexual shape dimorphism had been also described (Prenter, Montgomery & Elwood, 1995) using PCA based on linear combinations of linear size estimates (reviewed in Foellmer & Moya-Laraño, 2007), yielding to identify two main independent factors, one interpreted as “multidimensional size”, and another, orthogonal, interpreted as body condition or shape (Prenter, Montgomery & Elwood, 1995; Foellmer & Fairbairn, 2005a, 2005b). Not only spider shape resulting from the allometric growth of body parts had not yet been reported thus far; the fact that all such analyses were based on traditional morphometrics (i.e., linear measurements) implies that any such type of approach to spider morphological evolution would have been inevitably correlated to body mass and condition.

Patterns of shape differences in *D. merlini* suggest that sexual dimorphism results from divergent selection related to male and female reproductive roles and life styles. In our analyses, the main sex differences relate to the carapace-to-opisthosoma size, where females tend to exhibit disproportionally larger opisthosomas compared to males (Fig. 2). Additionally, sex differences in the allometric intercepts vary depending on whether we consider the body as a whole or only the carapace, and sex differences in body size and shape largely result from the relative growth of the opisthosoma. As spider opisthosoma is mostly related to egg production and storage (Foelix, 1996), fecundity selection arguably explains sex-diverging overall body morphologies in our case study species. Carapace morphologies and their sexual divergence also fit the expected considering sex-differential life-styles, particularly the different male and female activity patterns, where males are roving and females are permanent burrowers. Our results stress that male carapaces are clearly wider and more cephalically protruding, compared to those of the females. Several studies have indeed shown that mobility and competitive advantages drive the evolution of sex differences in spider carapace size (Moya-Laraño, Halaj & Wise, 2002; Foellmer & Fairbairn, 2005a, 2005b; Brandt & Andrade, 2007); more robust male carapaces could arguably be positively selected either by scramble or by contest competition, an hypothesis that requires further testing in field experiments.

*Donacosa merlini* morphology scales similarly between the sexes (i.e., slopes nearly equivalent for males and females), but differ in their intercepts (i.e., sexual differences are size-independent). The lack of sex differences between allometric slopes would argue against a role for sexual selection in the evolution of the observed sexual shape differences (Kodric-Brown, Sibly & Brown, 2006). However, sexual selection has been demonstrated to not always promote an increase in the allometric slope, but to lead instead to differences in the allometric intercepts (Bonduriansky, 2007; Shingleton & Frankino, 2012). On the other hand, sexually selected traits do not necessarily show allometry, whereas non-sexually selected traits often do (Shingleton & Frankino, 2012). Our results thus underscore that sexual selection might play an important role in the evolution of carapace shape differences.

These results also have important implications at the proximate level of this organism’s biology. First, the lack of sex differences in the allometric slopes and the existence of differences in the intercepts suggest that shape differences originate earlier in development. Stationary anatomical spider structures such as the carapace only expand at molting (Foelix, 1996), and sex differences in *D. merlini* life styles arise following the maturation molt, when males become wandering, indicating that carapace shape differences are likely to emerge at least following the penultimate molt. During development, arthropod SSD may result from sex differences in growth rate, development duration or size-dependent survival, though they usually result from an increased number of molts in one sex (Stillwell & Davidowitz, 2010). In order to achieve their larger carapaces, males might therefore undergo either an increased number of molts or an increased growth rate (i.e., heterochrony). A recent developmental model posits that differential trait growth may arise from differential sensitivity to environmental cues (i.e., plasticity) (Emlen et al., 2012), and males of a closely related species (*Lycosa hispanica*) are indeed known to respond more than females to variation in developmental conditions (Fernández-Montraveta & Moya-Laraño, 2007). Hence, male differences in carapace shape might result from a higher degree of male morphological plasticity in response to environmental variation. The alternative hypothesis that males undergo an increased number of molts during development is yet consistent with male delayed maturation (Fernández-Montraveta, Moya-Laraño & Cuadrado, 2004), and this question requires further testing.

Our results warn about previous common scientific wisdom on the evolution of spider SSD. Previously undetected sexually dimorphic carapace traits are far less noticeable than differences in the carapace-to-opisthosoma ratio, though they are definitely statistically significant (Fig. 2). Furthermore, a portion of this shape variation is allometric, probably entailing that only relatively larger spiders tend to exhibit more pronounced traits. Thus, it is unlikely that the observed differences could be detected in samples containing only small representatives of the species or reduced ranges of spider sizes. On the other hand, *D. merlini* could be considered either a female- or a male-biased sexually dimorphic species depending on the particular morphological trait measured: it shows the common Lycosidae pattern (F>M) on the basis of body size, yet it turns out to be male-biased and shows SSD reversal on the basis of the carapace size. This discrepancy is due to the fact that females exhibit comparatively larger opisthosomas compared to males, which show comparatively larger carapaces. Not surprisingly, the SSD pattern found when using carapace size is similar to that described on the basis of traditional metrics such as the carapace width (Foellmer & Moya-Laraño, 2007; Jakob, Marshall & Uetz, 1996). In all, SSD is a multifaceted phenomenon and our findings using shape analysis clearly warn about the limitations of single traits to make generalizations about this complex, yet crucial biological issue.

## Supplemental Information

10.7717/peerj.3617/supp-1 Supplemental Information 1 Raw data.

## Additional Information and Declarations

### Field Study Permissions

The following information was supplied relating to field study approvals (i.e., approving body and any reference numbers): Field experiments were approved by the Estación Biológica de Doñana (CSIC, project number 022/2007).

### Data Availability

The following information was supplied regarding data availability: The raw data have been supplied as Supplemental Dataset Files.
